# Spatial distribution and factors influencing modern contraceptive practice among tribal married women in India: evidence from National Family Health Survey 5 (2019–2021)

**DOI:** 10.1186/s12905-023-02454-5

**Published:** 2023-06-20

**Authors:** Sushree Nibedita Panda, Manish Barik, Ardhendu Sekhar Acharya, Srikanta Kanungo, Sanghamitra Pati

**Affiliations:** ICMR-Regional Medical Research Centre, Department of Health Research, Chandrasekharpur, Bhubaneswar, Odisha India

**Keywords:** Contraceptive, Tribals, India, Family planning

## Abstract

**Background:**

The unmet need for family planning has been a public health concern in developing countries, especially in the south-east region. In India, the expanding roles of women has led to a growing need for family planning and contraception. However, tribal women still struggle with reproductive and sexual health issues. Unfortunately, most tribal women are not informed about the potential health risks associated with contraceptive use, as service providers often neglect to provide this information. As a result, tribal women often suffer in silence, which can lead to serious health problems. Thus, the present study aimed to understand the patterns and factors associated with modern contraceptive usage as well as the district level variation in usage among tribal married women.

**Methods:**

We included 91,976 tribal married women participants aged 15 to 49 years from National Family Health Survey 5 conducted during the years 2019 to 2021. Descriptive statistics were used to compute the prevalence of modern contraceptive usage along with 95% confidence interval (CI) as a measure of uncertainty. The association between various socio-demographic predictors and modern contraceptive usage were assessed by multivariable logistic regression and presented as an adjusted odds ratio (AOR).

**Results:**

The overall prevalence of modern contraceptive practices was found to be 53% among tribal married women, which was below the national average. Sterilization was the most preferred method of modern contraceptive, whereas injectables were the least preferred method. More than 80% of the married women get the family planning information from the public health facility and health workers. Districts of eastern and north-eastern states have comparatively less modern contraceptive prevalence than central and southern states. Age, education, parity and access to media were significantly associated with the use of modern methods of contraception.

**Conclusion:**

Improving contraceptive use and reducing unmet needs for contraception among tribal women requires sustained efforts from healthcare workers, including Information Education and Communication (IEC) through mass media to raise awareness. A tailored family planning strategy is crucial to address the specific needs of tribal women at both the local and national levels, with adequate resources and monitoring for impact with this India can achieve Total Fertility Rate (TFR) 2.1 among tribals.

**Supplementary Information:**

The online version contains supplementary material available at 10.1186/s12905-023-02454-5.

## Background

Global data is replete with findings suggesting indigenous peoples have worse health and socioeconomic conditions than non-Indigenous populations [1], making them a vulnerable population. Indigenous groups in Low-medium income countries (LMICs) consistently have poorer health outcomes than non-indigenous groups; in particular, they carry a higher burden of infectious diseases and face elevated rates of child and maternal mortality [2]. Indigenous women face particular disadvantages due to the intersecting forces of indigeneity and gender, including a disproportionately higher burden of gender-based violence [3]. Around 8.6% of India’s population is made up of tribes (Scheduled Tribes) according to the census of 2011 while states like Madhya Pradesh, Jharkhand, Chhattisgarh, and Odisha have a higher concentration of tribal population [4]. Evidence from previous studies revealed that the tribal population suffers from poor health conditions, low literacy, poor economic and living conditions, and poor health-seeking behavior as compared to its non-tribal counterpart in India. There are problems of intra and interstate disparity with regard to socio-economical, demographic, and cultural diversities among tribals [5, 6].To bring the tribal population into the mainstream, several constitutional benefits have been initiated by the union government. Various welfare programs, reservation of seats in administration, government bodies, educational institutions, and so on for their upliftment but these measures could not be beneficial to the entire community [7].

The 2030 Agenda for Sustainable Development emphasizes the significance of monitoring contraceptive prevalence and the unmet need for family planning as key indicators for assessing improvements in reproductive health access. Various global and national initiatives have been launched to enhance the uptake of contraceptives, with the goal of achieving this target. However, as per National Family Health Survey NFHS-4 (2015–16), more than half of the currently married women aged 15–49 were not using any modern method of family planning in India where a similar trend was seen in tribal dominated states of India [8].

Among tribal populations, the use of contraception is considered a personal concern and it’s very challenging for them to surpass the family restrictions when it comes to modern contraception [7, 9, 10]. According to National Family Health Survey NFHS-5 (2019–21) report, India has reached the total fertility rate (TFR) target of 2.1, but TFR for tribal (Scheduled Tribe) was 2.5 compared to others with TFR of 1.8 [11]. Studies suggest that a larger part of the tribal population experience early marriage and contraceptive use is considerably low in spite of their high unmet need for family planning [7, 12]. Previous studies have also showed that among tribal communities the foremost reasons for not using contraception consist of fear of adverse effects, lack of knowledge, phobia of adverse health outcomes, religion, and past experiences [13, 14] All of these lead to early childbearing, shorter birth interval, and no healthy timing thereby causing mortality and morbidity among mothers and infants [15].

Thus, this paper aimed to estimate the prevalence of modern contraceptive practices, among tribal married women aged 15–49 years in India using nationally representative data from National Family Health Survey -5 (2019–2021) and to determine the socio- socio-demographic factors associated with the contraceptive practices. Additionally, the source of family planning information and understand the reason behind not using as well as district level variations.

## Method

### Overview of data

We used National Family Health Survey-5 (2019–2021) for the analysis, which provides vital information and statistics on household populations, housing features, basic socio-demographic and economic features of respondents, reproductive, maternal, infant and child mortality, nutrition, morbidity as well as adult health problems and domestic violence at the national, state and district level. NFHS was led by Ministry of Health and Family Welfare (MoHFW) and managed by the International Institute of Population Sciences (IIPS), Mumbai. NFHS-5 fieldwork for India has been carried out in two phases. Phase-I from 17 June 2019 to 30 January 2020 covering 17 states and 5 UTs and Phase-II from 2 January 2020 to 30 April 2021 covering 11 states and 3 UTs and collected data from 636,699 households, 724,115 women, and 101,839 men [11].

### Study population and sample size

**Fig. 1:**
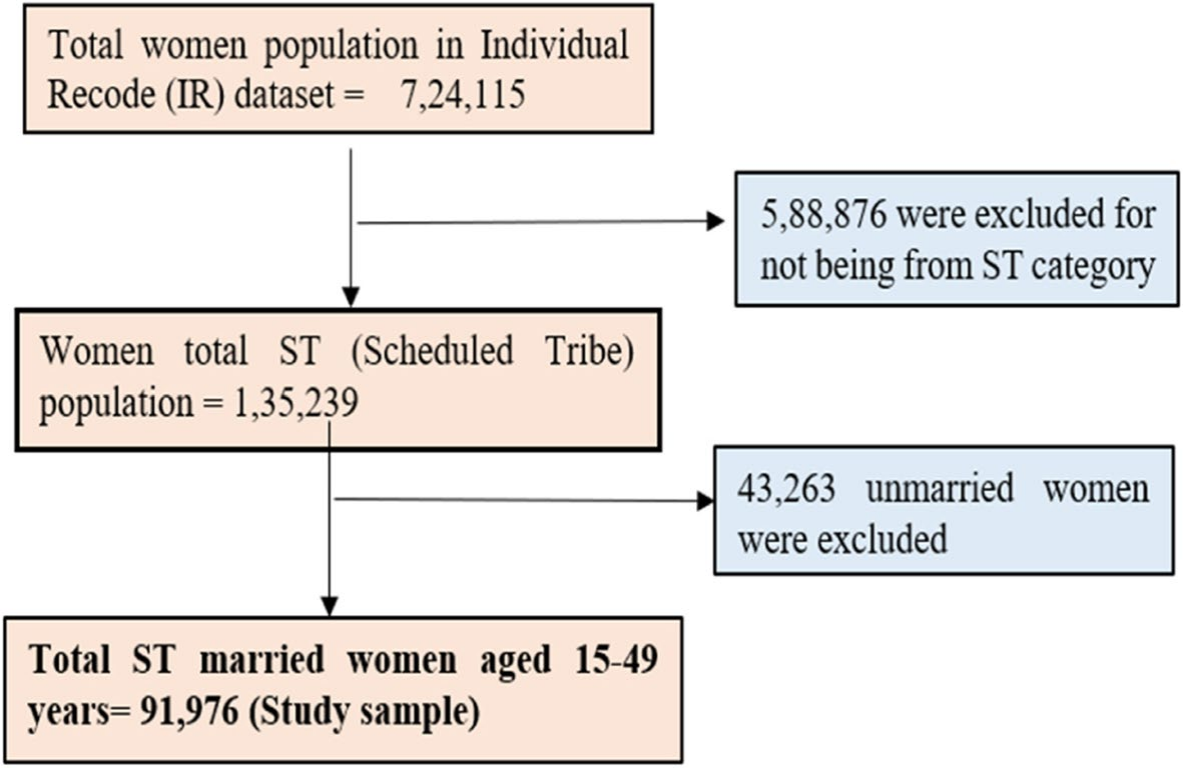
Selection criteria for study population

In this present study a total of 91,976 tribal married women aged 15–49 years were included for the analysis who were interviewed by individual schedule [16], the detailed sample derivation has been given in the Fig. 1.

### Outcome variable

The Individual questionnaire was administered by the field staffs for capturing several personal information. Participants were asked which type of contraceptive method they use, participants who have answered “Pill”, “Injectables”, “IUD”, “Condoms” and “sterilization (both male and female)” where defined as the “modern contraceptive method” to find out the prevalence of modern contraceptive method across different socio-demographic attributes.

### Explanatory variables

#### Individual characteristics

The independent variables were included by assembling previous pieces of literature. The included independent variables were socio-demographic factors (age, religion, place of residence, education, occupation, household wealth status, age at menarche, exposure to media and parity); and behavioural factors (cigarette/tobacco smoking).

In this study, the self-reported age of the women was grouped into the following age groups: “15–19 years”, “20–24 years”, “25–29 years”,”30–34 years”, “35–39 years”, “40–44 years” and “45–49 years”. Religion was classified as “Hindu”, “Muslim”, “Christian” and Sikh, Buddhist/neo-Buddhist, Jain, Jewish, Parsi/Zoroastrian, no religion was clubbed into “others”. The residence of the study participants was defined as “Rural” and “Urban”. Educational attainment was classified as “No formal education” (those who never attended school), “Primary” (up to 5^th^ grade), “Secondary” (up to grade 10), and “Higher” (above secondary level). The occupation was categorized as “Working” and “Not working”. In this study, Household wealth status was grouped as “Poorest” and “Poorer” “Middle”, and “Richer” and “Richest”.

Participant’s parity was categorized as per the children she has and labelled as “Nulliparity”, “Primiparity”, “ Multiparity” and “Grand multiparity”. Frequency of exposure to media variable was created using three variables such as “frequency of exposure to social media”, “frequency of exposure to TV”, and “frequency of exposure to radio” recoded as "not at all", "less than once a week" and "at least once a week". Tobacco use was categorized as “Yes” and “No”.

### Type of modern contraceptive method and source of family planning information

“Short acting reversible contraception” this includes Pill, Injectables and condoms, “Long-acting reversible contraception”: IUDs and the permanent method of contraception includes Female Sterilization and male Sterilization The sources of family planning information are Govt. health facility/ personnel, Private health facility/ personals, partner, friend/ related and other sources.

### Reasons for not using contraception

In this study participants were asked about why they are not using modern contraception, the answers were categorized as (1) Fertility-related: This includes “Infrequent sex, no sex”, “Menopause, hysterectomy”, “Sub-fecund, “in-fecund”, “Breastfeeding/Postpartum amenorrhoea”. (2) Opposition to use: This consisted of “Respondent opposed”, “Husband opposed”, “Others opposed” and “Religion prohibited”. (3) Lack of knowledge: “Knows no method”, “Knows no source/lack of access” (4) Method related: “Health concerns”, “Fear of side-effects”, “Cost too much”, “Interferes with body” “Inconvenient to use” and “Don’t like existing method”.

### Statistical analysis

Data were analysed using STATA version 17.0 (STATA Corp., Texas), Microsoft Office and Q GIS for the heat maps. Descriptive statistics were used to report the frequency and proportions of socio-demographic characteristics of the participants and modern contraceptive use. In order to examine the relationship between different participant characteristics and modern contraceptive use, binary logistic regression was performed. The statistically significant variables (p < 0.05) from the unadjusted model were used to run a multivariate logistic regression to examine the association between modern contraception use and various correlates, which was expressed as an adjusted odds ratio (AOR) with a 95% confidence interval (CI).Variation Inflation Factor (VIF) was checked in the regression model to detect multicollinearity among the explanatory variables. Sampling weights were taken into account during analysis for both descriptive and regression models.

### Ethical considerations

This study is based on secondary unnamed data obtained from NFHS 5 with no personal identifiers and hence there is no participant risk. The data were requested from DHS (Demographic health survey) through proper channel and appropriate permission was taken. The same have been properly acknowledged and referenced wherever required.

## Results

### Characteristics of study participants

This study included 91,976 currently tribal (ST) married women with an age range from 15 to 49 years old. Approximately more than 80% of the study population were Hindu and from rural areas. More than half (54.8%) of the study subjects were between the age group of 25–39 years. More than one-third of the tribal married women had no formal education followed by secondary education. This study estimated that 42% of the participants had more than two children. More than one third of the study population were exposed to media at least once a week. It was found that more than 50% of the tribal women had their menarche before the age of thirteen. The unemployment rate stood at 61% whereas more than 40% of the study population were in the poorest category Table 1.

**Table 1:** Socio-demographic characteristics of tribal married women in NFHS 5

Variables	Frequency	Percentage
**Age group**		
15–19 years	3,516	3.8%
20–24 years	14,229	15.4%
25–29 years	18,523	20.1%
30–34 years	16,558	18%
35–39 years	15,372	16.7%
40–44 years	12,108	13.1%
45–49 years	11,670	12.7%
**Respondent’s Education**		
Illiterate	38,492	41.8%
Primary	14,018	15.2%
Secondary	34,454	37.4%
Higher	5,010	5.4%
**Husband’s education**		
Illiterate	38,493	41.8%
Primary	14,018	15.2%
Secondary	34,454	37.5%
Higher	5,011	5.5%
**Religion**		
Hindu	80,044	87.1%
Muslim	1,875	2.04%
Christian	6,718	1.31%
Others	3,336	3.6%
**Wealth Index**		
Poorest	40,424	43.9%
Poorer	23,242	25.2%
Middle	14,535	15.8%
Richer	8,574	9.3%
Richest	5,198	5.6%
**Place of residence**		
Urban	11,123	12.09%
Rural	80,853	87.91%
**Parity**		
Nulliparity	9,257	10%
Primiparity	15,907	17.2%
Multiparity	28,101	30.5%
Grand multiparity	38,709	42.1%
**Frequency of exposure to media**		
Not at all	33,224	36.12%
Less than once a week	22,672	24.65%
At least once a week	36,080	39.23%
**Occupation**		
Employed	5,431	38.7%
Unemployed	8,607	61.3%
**Age at menarche**		
< 13 years	8,366	58.2%
> 13 years	5,988	41.8%
**Tobacco use**		
Yes	11,575	12.6%
No	80,400	87.4%

### Utilization of modern contraceptive practices among the tribal married women

Table 2 presents that around 53% of currently-married tribal women were using any modern method of contraception which is relatively lower compared to 67% of all Indian women using modern contraceptives. The majority of these tribal women preferred using female sterilisation (40.29%) followed by pills (5.03%). Injectables (0.49%) were reported to be least common method among tribal married women.

**Table 2:** Prevalence of different modern contraceptive methods of family planning among tribal women (15–49 years) in India

Contraceptives	N (%)	95% CI
**Modern method**	49,443 (53.7%)	53.4%-54.0%
**Short acting reversible contraception**		
Pill	4,629 (5.03%)	4.8%-5.2%
Injectables	446 (0.49%)	0.4%-0.6%
Condoms	4,557 (4.96%)	4.8%-5.1%
**Long-acting reversible contraception**		
IUDs	2,089 (2.27%)	2.1%-2.4%
**Permanent contraception**		
Female sterilization	37,055 (40.29%)	39.9%-40.6%
Male sterilization	652 (0.71%)	0.6%-0.7%

### Distribution of modern contraceptive prevalence rates among tribal married women by various background characteristics

**Fig. 2:**
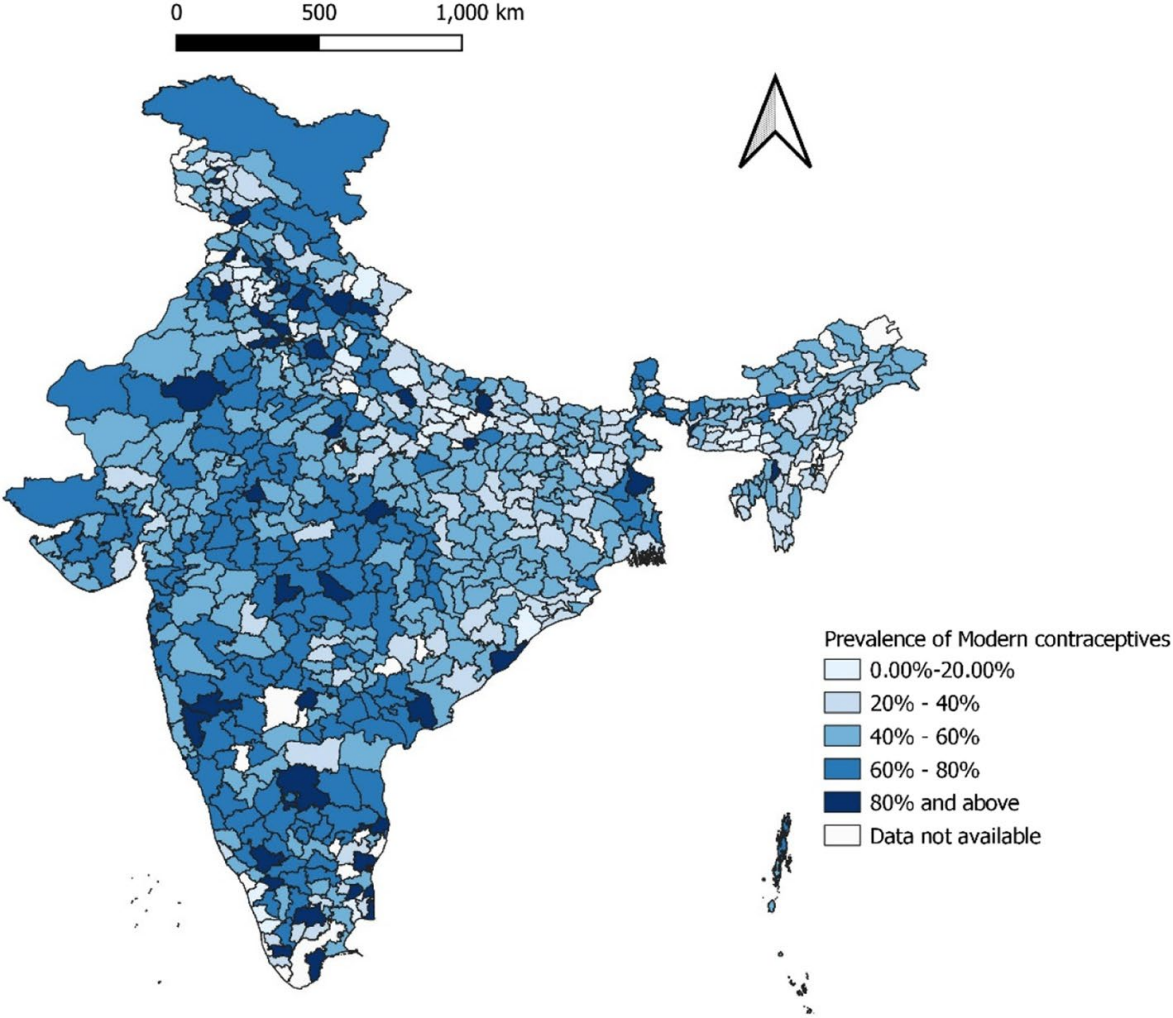
The district level prevalence of modern contraception practices among tribal married women

We found a greater prevalence of modern contraceptive usage among adults aged between 40 and 44 years, where almost 70% (68.7%) of the tribal female population were using any modern contraceptive methods. It is observed that higher levels of education are associated with lower rates of use of modern contraception whereas, a larger proportion of uneducated women were adopted modern methods of contraception. This study showed almost similar usage of modern contraceptive among participants belong to poorer and middle wealth status and prevalence was highest among richer class (58%) population. It was reported that tribal women whose husbands had a primary education were more likely to use modern contraceptive methods. In this study Hindu population had higher percentage of utilization of modern contraceptives. Participants from urban areas (56%) were more likely to use modern methods than their counterparts in rural areas. This study elucidated that the women with more than two children used modern contraceptives more frequently. The present study revealed that the higher percentage of modern contraceptive use was found among those who had exposure to media at least once a week (56.3%). Interestingly the findings showed that employed married tribal women (63.5%) were using modern contraceptives more than that of unemployed (49.1%). Modern contraceptive usage was higher (25.5%) among women who had early menarche (< 13 years). Almost similar prevalence was seen among the tobacco users and non-users Table 3. Figure 2 Illustrates the district level variation in prevalence of modern contraception use among tribal married women. The districts on eastern, central and north-eastern states have comparatively low prevalence of modern contraception compared to the southern and western India states.

**Table 3:** Prevalence of modern contraceptive use among married tribal women in India

Socio-demographic characteristics	Frequency (N)	Percentage (%)	95% Confidence interval (CI)
**Age group**			
15–19 years	471	13.4%	12.2%-14.5%
20–24 years	3,842	27%	26.2%-27.7%
25–29 years	8,675	46.8%	46.1%-47.5%
30–34 years	10,113	61%	60.3%-61.8%
35–39 years	10,331	67.2%	66.4%-67.9%
40–44 years	8,316	68.7%	67.8%-69.5%
45–49 years	7,691	65.9%	65.1%-66.7%
**Education**			
Illiterate	23,324	60.6%	60.1%-61%
Primary	7,892	56.3%	55.4%-57.1%
Secondary	16,161	47%	46.3%-47.4%
Higher	2,065	41.2%	39.8%-42.5%
**Husband’s education**			
Illiterate	2.096	57.4%	55.7%-59%
Primary	1,534	59.5%	57.5%-61.4%
Secondary	3,487	52.4%	51.2%-53.6%
Higher	536	48.6%	45.6%-51.6%
**Religion**			
Hindu	44,660	55.8%	55.4%-56.1%
Muslim	838	44.7%	42.4%-46.9%
Christian	2,480	36.9%	35.7%-38.0%
Others	1,481	44.4%	42.6%-46.1%
**Wealth Index**			
Poorest	20,233	50%	49.5%-50.5%
Poorer	13,057	56.1%	55.5%-56.8%
Middle	8,269	56.8%	56%-57.6%
Richer	4,974	58%	56.9%-59.1%
Richest	2,908	55.9%	54.5%-57.3%
**Place of residence**			
Urban	6,856	56.2%	55.3%-57%
Rural	42,586	53.3%	53%-53.7%
**Parity**			
Nulliparity	766	8.29%	7.7%-8.8%
Primiparity	4,675	29.3%	28.6%-30.1%
Multiparity	18,164	64.6%	64.1%-65.2%
Grand multiparity	25,835	66.7%	66.2%-67.2%
**Frequency of exposure to media**			
Not at all	17,858	51.1%	50.3%-51.8%
Less than once a week	10,920	53.6%	52.9%-54.3%
At least once a week	20,664	56.3%	55.8%-56.9%
**Occupation**			
Employed	3,450	63.5%	62.2%-64.8%
Unemployed	4,226	49.1%	48.1%-50.2%
**Age at menarche**			
< 13 years	2,137	25.5%	24.6%-26.4%
> 13 years	1,368	22.8%	21.7%-23.9%
**Tobacco use**			
Yes	6,497	56.1%	55.2%-57.1%
No	42,945	53.4%	53.1%-53.8%

### Multivariate regression analysis

Numerous characteristics, including the age of the tribal married women, religion, their education, wealth status, parity, and exposure to media had an influence on the modern contraceptive practice. Table 4 illustrates the odds for the use of modern contraceptive methods among tribal married women in India. Findings revealed that with the growing age the odds of modern contraception use are also rising, 40–44-year age group [AOR: 2.62 (2.11–3.26)] have the highest odds of modern contraception use. Participants with secondary and higher level of education are less likely to use modern contraceptive methods than the illiterate women. Women belonging to Hindu religion [AOR: 1.64(1.5–1.79)] were considerably more likely to use modern contraceptive methods as compared to other religion in this study. Modern methods of contraception were found to be higher among women who reported having multiparity [AOR: 15.07 (13.16–17.25)] or grand multiparity [AOR:14.88 (13.02–17.00)]. As the wealth quintiles reduced, the usage of modern contraceptives increased among the poorer population [AOR: 1.20(1.13–1.28)] than the wealthiest. Interestingly, tribal women who were exposed to media at least once a week [AOR: 1.38 (1.29–1.37)] evolved as the strongest predictor of modern contraception use followed by less than week [AOR: 1.24 (1.16–1.32)].

**Table 4:** Multivariate regression analysis depicting association of socio-demographic attributes with modern contraceptive use

Socio-demographic variables	Adjusted OR	95% CI	*p*-value
**Age**			
15–19 years	Ref		
20–24 years	0.94	0.76–1.17	0.605
25–29 years	1.36	1.10–1.68	0.003
30–34 years	2.07	1.67–2.56	0.000
35–39 years	2.57	2.08–3.19	0.000
40–44 years	2.62	2.11–3.26	0.000
45–49 years	2.27	1.82–2.82	0.000
**Education**			
Illiterate	Ref		
Primary	0.97	0.90–1.04	0.502
Secondary	0.91	0.85–0.97	0.005
Higher	0.80	0.66–097	0.028
**Religion**			
Hindu	1.64	1.5–1.79	0.000
Muslim	0.84	0.70–1.00	0.053
Christian	Ref		
Others	1.40	1.23–1.60	0.000
**Wealth Index**			
Poorest	Ref		
Poorer	1.20	1.13–1.28	0.000
Middle	1.18	1.09–1.28	0.000
Richer	1.18	1.05–1.32	0.005
Richest	1.05	0.88–1.26	0.544
**Place of residence**			
Urban	1.04	0.94–1.16	0.400
Rural	Ref		
**Parity**			
Nulliparity	Ref		
Primiparity	4.47	3.88–5.14	0.000
Multiparity	15.07	13.16–17.25	0.000
Grand multiparity	14.88	13.02–17.00	0.000
**Frequency of exposure to media**			
Not at all	Ref		
Less than once a week	1.24	1.16–1.32	0.000
At least once a week	1.38	1.29–1.47	0.000
**Tobacco use**			
Yes	1.00	0.94–1.07	0.783
No	Ref		

### Major reasons for not using modern contraception

Supplementary Table 1 describes the major reasons provided by the study participants for not practicing modern contraception. The most prevalent reason for the disuse of contraception was “menopause or hysterectomy” followed by “husband opposition”. But the prevalence of each reason varied substantially. Tribal married women reported non-use due to “Sub-fecund or in-fecund” which stood at 7.11%. Frequency of non-use due to “infrequent sex or no sex”, “respondent opposed”, and “health concerns” were almost similar. This study showed that some participants did not like existing methods which was one of the reasons for not using modern contraceptives. We found that “Fear of side-effects” around 4.75%, a reason for non-utilization of modern contraception. Other reasons that contributed to non-use of modern contraception were “Breastfeeding/Postpartum amenorrhoea”, “Others opposed”, “Religion-prohibited”, “high cost” and lack of knowledge.

### Source of modern contraceptives usage

**Fig. 3:**
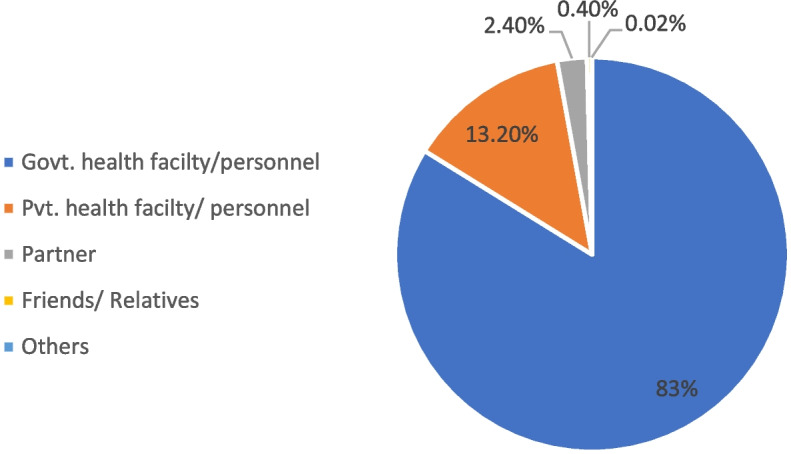
The main sources of modern contraceptives usage among tribal married women

The current study indicated that tribal married women received modern contraceptives from number of sources. More than 80% tribal married women reported of receiving modern contraceptives from Government Health facility/ personnel followed by private health facility/personnel. Only 2.4% mentioned their partner as one of the sources of modern contraceptives followed by friends/relative Fig. 3.

## Discussion

The present study's main focus was on the use of modern contraceptives by tribal married women in India, where their fertility rate was relatively high. Although not new, the use of methods of contraception among tribal people is a rather unexplored topic. For several years, this challenge has been the primary subject of concern. There are few surveys that detail how diverse the country's tribes are in terms of their use of family planning. The NFHS survey, which includes all districts all over the country, provides an exceptional opportunity to analyse the district wise distribution of contraceptive practice among tribal women. Some states have hilly landscape and communities are dispersed across a large area, which poses a number of significant issues for the delivery of family planning and reproductive health services [17]. Studies carried out on Indian population also confirms these findings [18, 19]

Awareness has a significant role to play in incorporating positive attitude among tribal married women to adopt modern contraception. The current study revealed that more than 50% of the tribal married women used modern methods for family planning. This is lesser than that the national average of 67% [11]. Among the participants who utilized family planning methods, more than 40% of the study participants were using a non-reversible method. The fact that female sterilisation accounted for more than three-fourths of all contraception use, which indicates that tribal women were primarily employing family planning techniques to reduce their family size, and child spacing was largely overlooked. The tribes' poor economic conditions and the financial incentives linked with sterilisation contributed to the higher acceptability of sterilization among them [20]. According to prior studies, the major reason for sterilisation is that it needs one-time motivation, whereas the encouragement for spacing methods necessitates deliberate attempts [21]. However, the limited access of mass media and low literacy rates among tribal populations may contribute to a lack of awareness about temporary contraceptive methods**.** Following sterilization as a modern method for contraception use of pills were second highest prevalent method. Studies conducted in different regions of world also confirm the same [22–24].

The age of tribal women has emerged as a significant factor in predicting their usage of contraceptives. Women approaching menopause have a higher rate of contraceptive utilization, which is in line with the previous studies conducted in Nepal, United States of America (USA), Bangladesh and Iran [23–25]. This study's results indicate that in order to avoid unintended pregnancies, individuals under 35 years should be the focus. Policy makers must implement educational programs specifically for this age range to emphasize the significance of utilizing contraceptives and the risks of unintended pregnancy. The findings of the study highlight the complex relationship between education and contraceptive use, particularly in tribal populations. Despite being disadvantaged and facing various socio-economic challenges, it was found that illiterate women were using modern contraceptive methods more frequently compared to their literate counterparts, the possible reason could be the higher sterilization rate and the financial incentive associated with it. However previous studies have suggested that the higher education level is associated with the modern contraceptive use [9, 22, 26]. Tribal women following Hindu religion were associated with the increased modern contraceptive use, however previous studies also confirms the same [27, 28]

In this study, it was discovered that tribal women's wealth status was a significant indicator of their usage of contraception. Wealth can also influence cultural and social norms, as well as personal attitudes and beliefs towards family planning. Women from higher wealth quintiles may be more likely to have greater exposure to information and resources about family planning, and be able to form informed decisions about their reproductive health [29, 30]. However, the interaction between wealth status and contraceptive use was not significant for women in the richest quintile. Previous lot of studies also are in line with this [31–33].

The analysis showed that the odds of contraceptive practice increasing with increase in amount of parity. Studies carried out in different regions also support the finding [23, 34], Prior evidence showed that partners, peers, and elders have influence on decision to use contraception among women which can vary based on the number of children she has had [35].

In this study tribal women having high mass media exposure is associated with the increased modern contraceptive practice which are in concordance with the previous literature [7]. Mass media exposure can have a complex impact on promoting contraceptive use among tribal women. While mass media can raise awareness and dispel myths surrounding contraception, it is also important to recognize the cultural and social norms of tribal communities which can shape attitudes towards contraceptive use [36]. In some instances, traditional beliefs or practices may not align with the messages conveyed through mass media, hindering its effectiveness in promoting contraceptive use. To increase contraceptive use among tribal women, it may be necessary to adapt mass media messages to their unique cultural and social context, taking into consideration their values, beliefs, and practices [37, 38]. After achieving independence, the Indian government has implemented numerous initiatives to control the growth rate, which is contributing to the rising rates of female sterilisation. In the 1950s, India was the first country to introduce a family planning programme that offered married women a variety of contraceptive options [39]. Following that, the government promptly adopted numerous policies. With the purpose of increasing mother and child care services (MCH) in addition to the prevalence rate of modern contraception (mCPR) in the nation, India launched the spectacular National Rural Health Mission (NRHM) during the 2005–2006 in rural areas and later in 2011 in urban areas [33, 40].

Additionally, community engagement and involvement are vital in establishing trust and fostering open dialogue about contraception. This, combined with access to contraceptive services, can help to promote contraceptive use and enhance the health and well-being of tribal women. By collaborating with tribal communities and utilizing the power of mass media, it may be possible to effectively encourage contraceptive use.

### Strength and limitations

Our study utilized a representative sample of the tribal population in India drawn from nationwide data, allowing us to perform a comprehensive analysis that represents the entire country. To our knowledge, this is the first study that sheds light on the connection between various predictors and modern contraceptive use in the tribal married women population in India. This study relied on self-reported data, which may be subject to recall bias or social desirability bias. It is also important to consider the potential influence of cultural and social norms on contraceptive use, which may not be fully captured in the data. These limitations suggest that the results should be interpreted with caution and further qualitative research is needed to better understand the complex factors such as culture, beliefs and customs that influence contraceptive use among tribal married women.

## Conclusion

The improvement of the overall prevalence of contraceptive usage and the resolution of unmet needs for contraception need a sustained and ongoing effort from healthcare professionals to provide excellent care to the beneficiaries. One crucial aspect of this effort is the emphasis on IEC (Information, Education, and Communication) initiatives through mass media, which can help to raise awareness among tribal married women. To effectively address this issue, it is also crucial to develop a comprehensive family planning strategy that is tailored to the specific needs of tribal women, both at the micro and macro levels. This strategy should then be implemented with adequate resource allocation and monitored and evaluated to assess its impact, especially with regards to equity. The ultimate goal is to achieve a TFR (total fertility rate) of 2.1 among tribals. By making a concerted effort, health workers, policymakers, and communities can play a significant role in improving the contraceptive prevalence rate among tribal married women and promoting better health and well-being for these communities.

## Supplementary Information

**Additional file 1:** **Supplementary Table 1.** Reasons for not usingmodern contraception among tribal married women in India.

## Data Availability

All data are publicly available and can be accessed through The DHS Program, https://dhsprogram.com/data/.
