# Supplementary material for: Spatial distribution and factors influencing modern contraceptive practice among tribal married women in India: evidence from National Family Health Survey 5 (2019–2021)
Source: BMC Womens Health. 2023 Jun 20;23:318. doi: 10.1186/s12905-023-02454-5 (PMC10281003; doi:10.1186/s12905-023-02454-5)
Supplement: Supplementary file 1 — Additional file 1: Supplementary Table 1. Reasons for not usingmodern contraception among tribal married women in India. [file 12905_2023_2454_MOESM1_ESM.docx]

Supplementary table 1 : Reasons for not using modern contraception among tribal married women in India

| **Main reasons** | **N,%** |
| --- | --- |
| **Fertility-related** | |
| Infrequent sex, no sex | 4,067(4.42%) |
| Menopause, hysterectomy | 2,184 (14.68%) |
| Sub-fecund, in-fecund | 1,057(7.11%) |
| Breastfeeding/Postpartum amenorrhoea | 2,238(2.43%) |
| **Opposition to use** | |
| Respondent opposed | 751(5.05%) |
| Husband opposed | 1,563(10.51$%) |
| Others opposed | 88(0.59%) |
| Religion prohibited | 46(0.32%) |
| **Lack of knowledge** | |
| Knows no method | 95(0.64%) |
| Knows no source/lack of access | 143(0.97%) |
| **Method-related** | |
| Health concerns | NA |
| Fear of side-effects | 706(4.75%) |
| Cost too much | 445(3%) |
| Interferes with body | 110(0.74%) |
| Inconvenient to use | 157(1.06%) |
| Don’t like existing method | 554(3.72%) |
